# Stemness related lncRNAs signature for the prognosis and tumor immune microenvironment of ccRCC patients

**DOI:** 10.1186/s12920-024-01920-9

**Published:** 2024-05-31

**Authors:** Mengjiao Zhang, Jiqiang Zhang, Xuemei Liang, Ming Zhang

**Affiliations:** 1 Department of Geriatric Medicine, The Affiliated Hospital of Southwest Medical University, Luzhou, 646000 China; 2 Department of Orthopedics/Sports Medicine Center, Southwest Hospital, Army Medical University, Chongqing, 400038 China; 3 Department of Respiratory and Critical Care Medicine, The Affiliated Hospital of Southwest Medical University, Luzhou, 646000 China

**Keywords:** Cancer stem cells, Clear cell renal cell carcinoma, Long non-coding RNAs, Tumor immune microenvironment, Prognosis

## Abstract

Long non-coding RNAs (lncRNAs) and cancer stem cells (CSCs) are crucial for the growth, migration, recurrence, and medication resistance of tumors. However, the impact of lncRNAs related to stemness on the outcome and tumor immune microenvironment (TIME) in clear cell renal cell carcinoma (ccRCC) is still unclear. In this study, we aimed to predict the outcome and TIME of ccRCC by constructing a stem related lncRNAs (SRlncRNAs) signature. We firstly downloaded ccRCC patients’ clinical data and RNA sequencing data from UCSC and TCGA databases, and abtained the differentially expressed lncRNAs highly correlated with stem index in ccRCC through gene expression differential analysis and Pearson correlation analysis. Then, we selected suitable SRlncRNAs for constructing a prognostic signature of ccRCC patients by LASSO Cox regression. Further, we used nomogram and Kaplan Meier curves to evaluate the SRlncRNA signature for the prognose in ccRCC. At last, we used ssGSEA and GSVA to evaluate the correlation between the SRlncRNAs signature and TIME in ccRCC. Finally, We obtained a signtaure based on six SRlncRNAs, which are correlated with TIME and can effectively predict the ccRCC patients’ prognosis. The SRlncRNAs signature may be a noval prognostic indicator in ccRCC.

## Introduction

Clear cell renal cell carcinoma (ccRCC) is the most common subtype of renal cell carcinoma, making up between 75% and 80% of all cases [1]. At the moment, the annual incidence rate of ccRCC is increasing. The absence of symptoms and indications in individuals with initial stage ccRCC results in a significant proportion of patients already experiencing tumor metastasis upon diagnosis, consequently resulting in an unfavorable prognosis. Despite the favorable outcomes attained in tumor therapy with targeted treatment and immunotherapy, ccRCC remains a challenging condition for advanced patients, with a mere 13% five-year survival rate [2]. Hence, discovering novel biomarkers is of utmost significance for ccRCC individuals’ diagnosis, management and prognosis.

Cancer stem cells (CSCs) participate in tumor genesis and regeneration, and are the foundation of tumor occurrence and development, which and have the ability to self-renew [3, 4]. Researches have indicated that CSCs have a strong correlation with invasion and metastasis of tumors [5–8]. Meanwhile, tumor cells can generate new CSCs through reprogramming mechanisms, and these CSCs can exhibit different characteristics. The diversity of CSCs makes tumors resistant to anticancer therapies [9, 10]. Therefore, studying tumor stem cells holds significant clinical importance in treatment of tumors, including ccRCC. Tumor stemness is a term used to describe the functional characteristics of tumor stem cells, including their capacity for differentiation and self-renewal. Acquisition of stemness is a critical element in malignancies’ formation and progression, and it can be said that cancer evolves is the process by which tumor cells acquire stemness [11, 12]. The mRNAsi, which is based on the expression of mRNA, serves as a significant gauge that mirrors the gene expression traits of stem cells. The mRNAsi is obtained through a machine-learning algorithm of mRNA expression related to stemness in cells, high mRNAsi is associated with active biological processes and obvious dedifferentiation in CSCs [13]. LncRNA is abnormally expressed in various cancers, it has be found that multiple lncRNAs can participate in metastasis, resistance to drugs, and recurrence of cancer by regulating the stemness of CSCs [14–16]. Studies have shown that the stem related lncRNAs (SRlncRNAs) were strongly correlated with the prognosis of individuals [17, 18], however, Whether the SRlncRNAs would affect the prognosis of ccRCC is still unknown.

In this research, we selected 6 SRlncRNAs to build a predictive pattern for individuals with ccRCC. Next, We evaluated the signature’s prognostic predictive capacity and examined its association with the immune microenvironment of ccRCC.

## Material and method

### Collection and preparation of data

We downloaded ccRCC patients’ clinical information and RNA sequencing data from UCSC (https://xenabrowser.net, retrieved on August 15, 2023) and TCGA (https://portal.gdc.cancer.gov, retrieved on May 9, 2023), including 72 kidney normal tissues, 541 ccRCC tumor samples and 532 ccRCC patients’ clinical data. The inclusion criteria for the study: The overall survival of patients is not 0, and tumor samples from patients are of RNA sequencing data. 528 ccRCC patients were included in the study. We downloaded human stem cell data using the “synapser” R package, and constructed a model for computing mRNAsi by using the “gelnet” R package. Next, we utilized the model to compute the mRNAsi of every tumor sample.

### Determination of the SRlncRNAs related to prognosis

Firstly, we utilized the “Deseq2” R software package to acquire differentially expressed lncRNAs in ccRCC tumor tissue compared normal kidney tissue(padj = 0.05, log2(foldChange) = 1) [19]. Subsequently, we employed Pearson correlation analysis to acquire SRlncRNAs that had strong association with mRNAsi (Pearson correlation coefficient is at least 0.4). We used “Venn” R package to obtain differentially expressed SRlncRNAs, and used univariate COX regression analysis for acquiring the differentially expressed SRLncRNAs closly associated with ccRCC patients’ prognosis.

### Construction of the SRlncRNAs signature in ccRCC

We randomly divided 528 patients into two groups (training and testing). LASSO COX regression was applied to selected lncRNAs for constructing a prognostic SRlncRNAs signature and calculating its coefficients in the training group. The software package ”glmnet” was used to perform LASSO COX regression. The SRlncRNAs signature was used to compute all patients’ risk scores as follow formula: Risk score=**∑** expression of lncRNA * coefficient. Subsequently, we computed all patients’ risk scores, and employed Kaplan-Meier (KM) and Receiver Operating Characteristic (ROC) curves to examine the relationship between risk scores and ccRCC patients’ prognosis. In additon, we evaluated the predictive ability of this sigature for ccRCC patients’ prognosis by utilizing COX regression analysis and nomograms.

### The SRlncRNAs signature in different clinical subgroups

Different clinical subgroups’ risk scores for patients with ccRCC were compared. The relationship between risk scores and ccRCC patients’ overall survival (OS) was evaluated using KM curves.

### The relationship between the SRlncRNAs signature and biology pathways

The median risk scores were used to divive patients into two groups(high risk and low risk). Then, We used “Deseq2” to analyze differences in gene expression between the two groups. At last, we perfomed gene set enrichment analysis (GSEA) based on the fold change of genes to analyze the relationship between SRlncRNAs signature and biology pathways. GSEA was performed utilizing the R package ‘clusterProfiler’ [20]. The reference was the gene set “c2. cp. kegg. v2023.1. Hs. symbols” sourced from Molecular Signatures Database (https://www.gsea-msigdb.org/gsea/msigdb, accessed on August 17, 2023). The statistical significance is indicated by *p* < 0.05 and false discovery rate (FDR <0.25.

### The SRlncRNAs signature and TIME relationship in ccRCC

Initially, we employed “ESTIMATE” R package to e evaluate the correlation between risk scores and “ESTIMATE” scores (matrix, immunity, and estimate) in ccRCC [21]. Then, We applied a CIBERSORT algorithmobtain to obtain immunocytes infiltration score of tumor tissues [22]. Subsequently, we compared the immune checkpoint expression and the immune cell infiltration scores between high and low risk groups. At last, we utilized “GSVA” R package [23] to conduct a single sample GSEA (ssGSEA) for finding the differences between the two groups’ immune-related pathways.

### Statistic analysis

We performed statistical analysis using R 4.13 and SPSS25. Two samples were compared using t-test. *P* ≤ 0.05 means statistical significance.

## Result

### Identification of SRlncRNAs associated with ccRCC patients’ prognosis

**Fig. 1:**
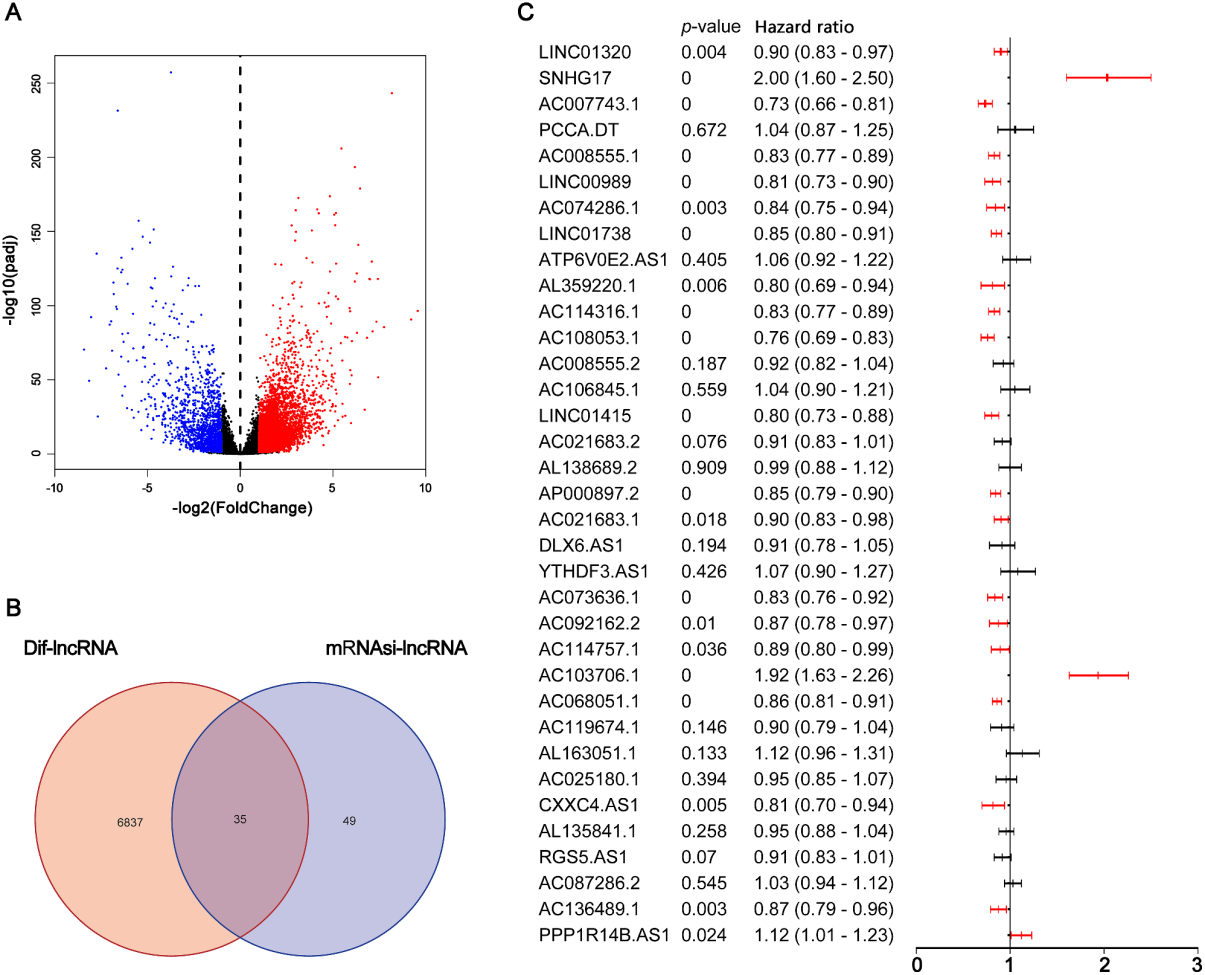
SRLncRNAs closely associated with ccRCC patients’ prognose. (**A**) The volcano plot demonstrated how the lncRNAs expressed differently in the surrounding normal kidney tissue and the ccRCC tumor tissue. (**B**) The Venn plot showed differentially expressed SRLncRNAs. (**C**) Univariate COX regression analysis indicated the number of differentially expressed SRLncRNAs closely associated with ccRCC patients’ prognosis

There were 6866 differentially expressed lncRNAs between ccRCC tumor tissue and normal kidney tissue, including 5431 upregulated and 1435 downregulated lncRNA in tumor tissues (Fig. 1A). There were 84 SRlncRNAs and 35 differentially expressed SRlncRNAs (Fig. 1B). There were 21 differentially expressed SRlncRNAs in relation to OS of ccRCC patients in univariate COX regression analysis (*p* < 0.05) (Fig. 1C).

### Construction and validation of the SRlncRNAs signature

**Fig. 2:**
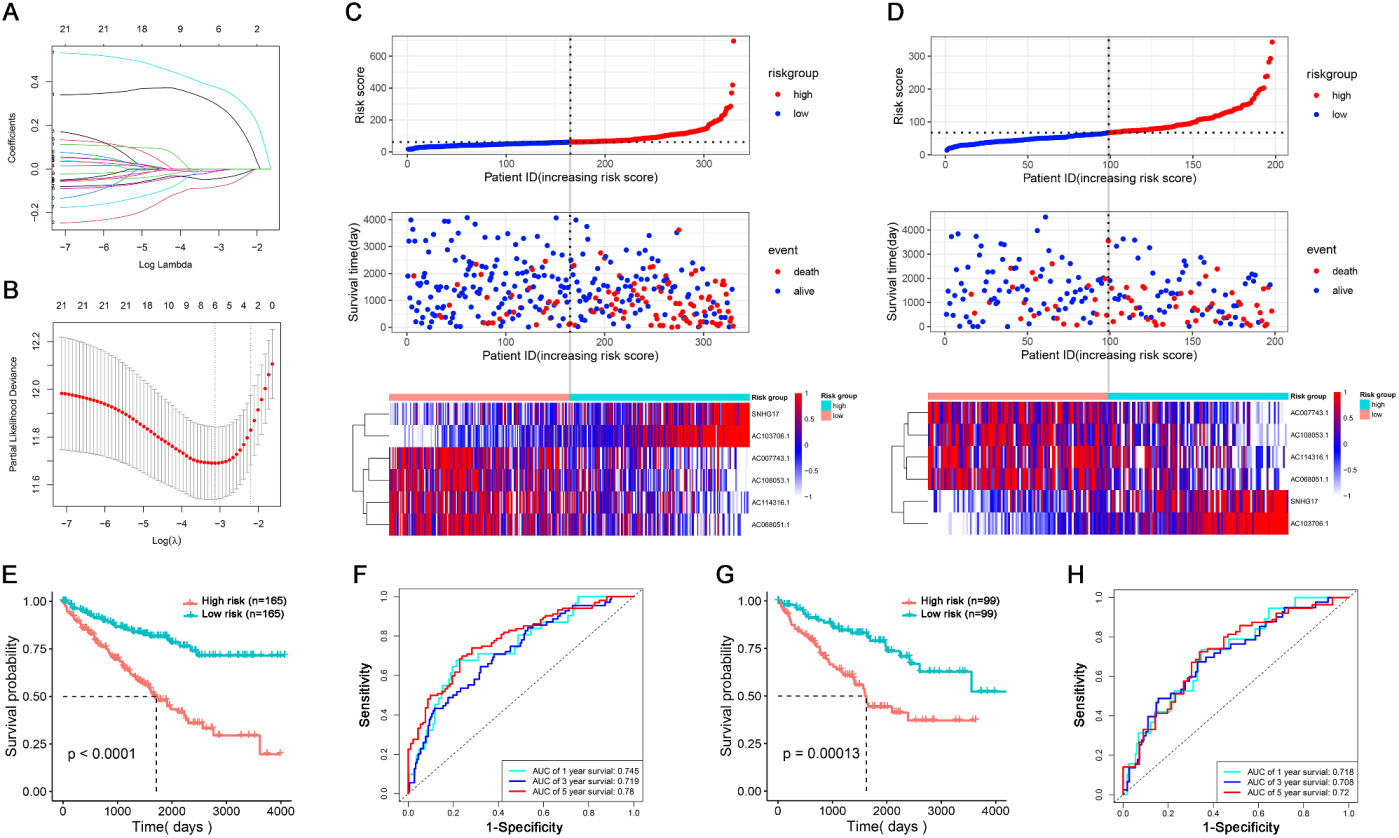
A SRLncRNAs signature of prognosis in ccRCC patients. (**A**) SRlncRNAs coefficient distribution plot. (**B**) Partial likelihood deviation plot. (**C**-**D**) The training and testing sets’ risk groups, population distribution and SRlncRNAs’ expression. (**E** and **G**) In both the training and testing sets, Kaplan-Meier curves demonstrated that patients’ survival probabilities were lower in high than low risk groups. (**F** and **H**) ROC curves both showed the SRlncRNAs signature was highly sensitive and specific to predict ccRCC patients’ survival probability both in training and testing sets

Random assignment was used to divide the 528 patients into two groups (training and testing), the training and testing groups respectively included 330 and 198 patients. (Table 1). In the training group, LASSO Cox regression analysis was utilized to select SRlncRNAs and calculate coefficients of the model. When lambda was set as the minimum value (lambda.min = 0.0438), there were 6 SRlncRNAs be chosed to construct a prognostic signature (Risk score =“SNHG17”×0.3036-“AC007743.1"×0.0827-“AC114316.1"×0.0129-“AC108053.1"×0.0454+"AC103706.1"×0.3787-“AC068051.1"×0.0085) (Fig. 2A, B). The risk score for each patient was determined by applying the prognostic signature. The patients were categorized into two groups (high risk and low risk) based on the median of the risk scores. Both in the training and testing group, more people died in high than low risk group (Fig. 2C and D). Compared to low-risk patients, the training group’s high-risk patients had a reduced survival probability. (*p* < 0.0001) (Fig. 2E), the SRlncRNAs signature was highly sensitive and specific to predict ccRCC patients’ survival probability, AUCs were 0.745 for 1 year survival, 0.717 for 2 years survival, and 0.78 for 5 years survival (Fig. 2F). Additionally, the testing group’s high-risk patients had a poorer survival probability than its low-risk counterparts. (*p* < 0.001) (Fig. 2G), the SRlncRNAs signature was highly sensitive and specific to predict ccRCC patients’ survival probability, AUCs were 0.718 for 1 year survival, 0.708 for 2 years survival, and 0.72 for 5 years survival (Fig. 2H).

**Table 1:** Clinical characteristics of ccRCC patients in the training and testing sets

Clinical factor	Training set (*n* = 330)	Testing set (*n* = 198)	Overall (*n* = 528)
Survival time (day)	1341.63 ± 976.66	1344.10 ± 983.03	1342.56 ± 978.13
Status			
live	223	132	355
Dead	107	66	173
Gender			
Female	118	67	185
Male	212	131	343
Age (year)			
< 60	149	96	245
≥ 60	181	102	283
Tumor grade			
1	6	7	13
2	140	176	228
3	128	76	204
4	51	24	75
Unknown	5	3	8
T Stage			
T1	159	109	268
T2	46	23	69
T3	116	64	180
T4	9	2	11
Unknown	0	0	
N Stage			
N0	143	96	239
N1	10	6	16
Unknown	177	96	273
M Stage			
M0	264	155	419
M1	47	32	79
Unknown	19	11	30
Tumor Stage			
I	155	107	262
II	40	17	57
III	84	39	123
IV	49	34	83
Unknown	2	1	3

### The SRlncRNAs signature was closely associated with ccRCC patients’ prognosis

Univariate Cox regression analysis showed the gender was not significantly related with patients’ OS (*p* > 0.05). Tumor grade, tumor stage, age and risk score were all unfavorable prognostic factors for ccRCC patients (*p* < 0.001). ccRCC patients’ prognosis were independently influenced by age, tumor stage, and risk score. (*p* < 0.001) (Table 2).

**Table 2:** Univariate and multivariate Cox regression analysis with OS of ccRCC patients

	Univariate analysis			Multivariate analysis		
Covariates	HR	95% CI	P‑value	HR	95% CI	P‑value
Gender						
(male vs. female)	0.951	0.697-1. 296	0.749			
Age (year)	1.031	1.018–1.044	< 0.001*	1.028	1.014–1.043	< 0.001*
Tumor stage	1.903	1.667–2.172	< 0.001*	1.637	1.407–1.905	< 0.001*
Tumor grade	2.302	1.879–2.818	< 0.001*	1.194	0.940–1.516	0.147
Risk score	3.319	2.588–4.257	< 0.001*	2.239	1.696–2.956	< 0.001*

* *p* < 0.05

**Fig. 3:**
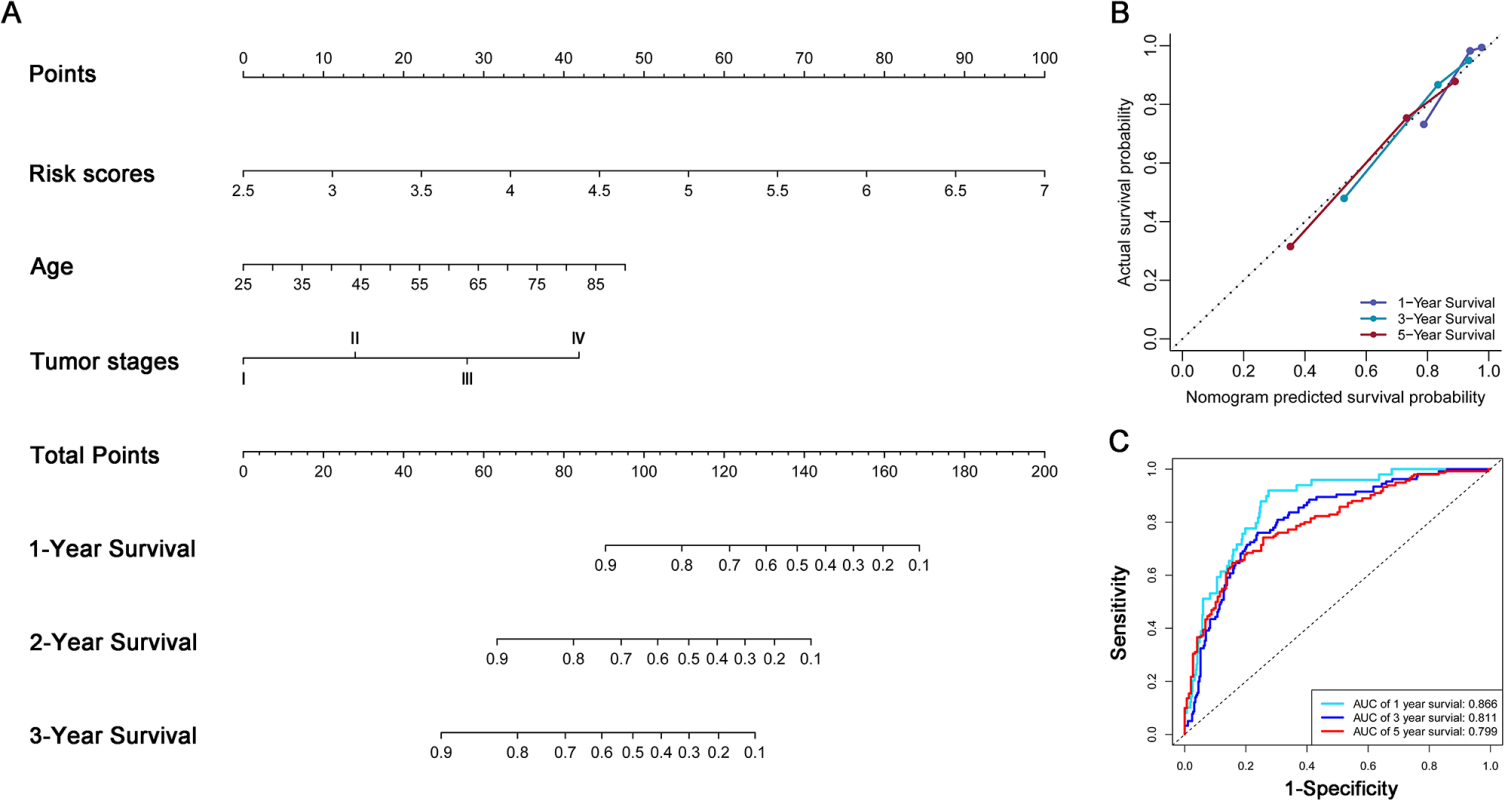
A nomogram for ccRCC patients’ prognosis. (**A**) A nomogram constructed of the tumor stage, age and risk scores. (**B**) Calibration curves indicated the nomogram was of high accuracy. (**C**) ROC curves indicated the nomogram was of good specificity and sensitivity

Subsequently, we utilized the three independent impact factors to build a nomogram for patients’ prognosis. It was observed that the impact of risk scores on patients’ survival was more significant than the age and tumor stage (Fig. 3A). Nomogram predictions were close to real values for 1, 2 and 3-year survival (Fig. 3B). The nomogram was highly sensitive and specific for predicting patients’ survival, AUC values of 1, 2, 3-year survival were respectively 0.886, 0.811, and 0.799 (Fig. 3C). This nomogram’ C-index is 0.782 with a confidence interval of 0.765–0.799. The C-index of the nomogram constructed of age and tumor stage is 0.755 with a confidence interval of 0.737–0.773. We can see that the risk score can increase the nomogram’s accuracy in predicting patients’ prognoses.

**Fig. 4:**
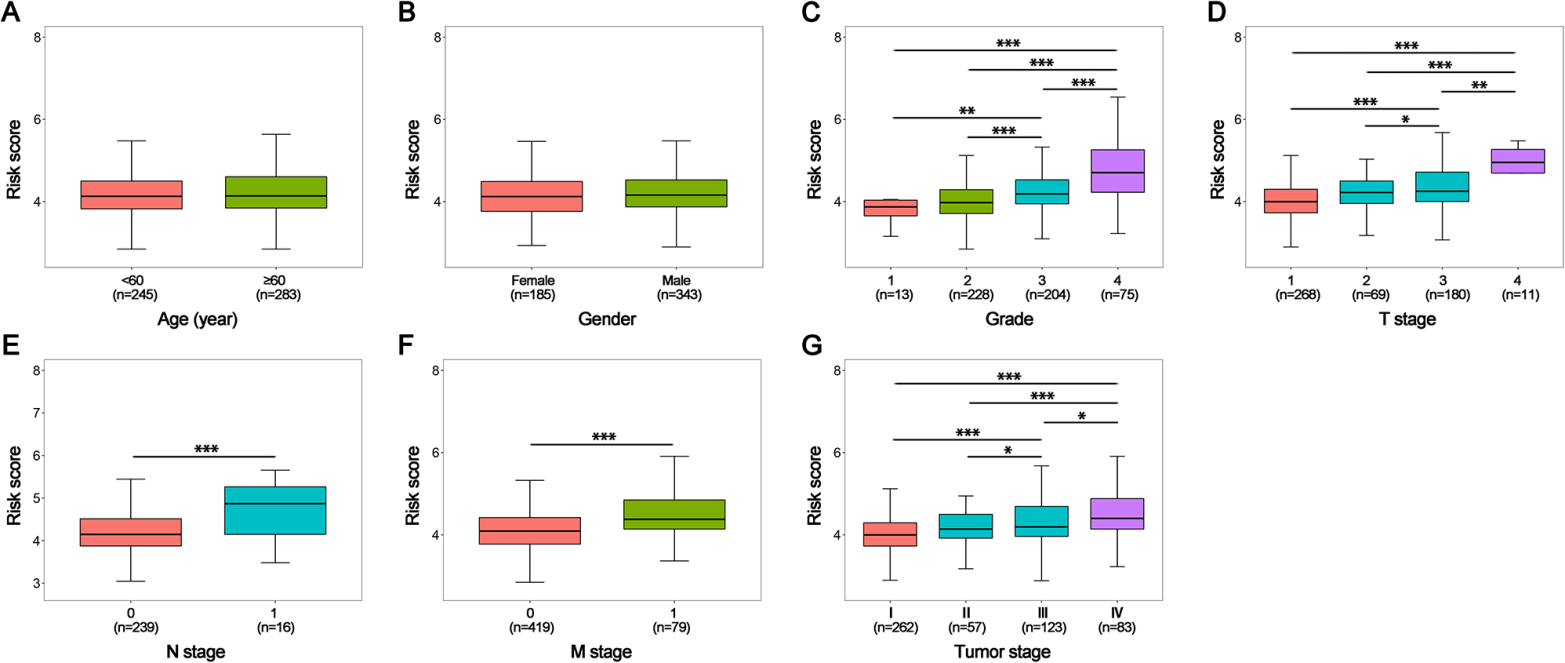
The risk scores in different clinical subgroups in ccRCC. (**A**) Age; (**B**) Gender; (**C**) Tumor grade; (**D**) T (tumor size) stage; (**E**) N (lymph node metastasis) stage; (**F**) M (distant metastasis) stage; (**G**) Tumor stage. * *p* < 0.05, * * *p* < 0.01, * * * *p* < 0.001

We performed the comparison of risk scores of patients in clinical subgroups. The difference of risk scores in two age subgroups was not significant, as well as in two gender subgroups (*p* > 0.05) (Fig. 4A and B). Across all tumor grade groups, the high grade groups had greater risk scores, the difference between the 1 and 2 grade groups had no significance (*p* > 0.05), but not in others(*p* < 0.05) (Fig. 4C). Both in the T stage and tumor stage groups, the risk scores were high in high stages, the difference between the 1 and 2 grade groups had no significance (*p* > 0.05), but not in others (*p* < 0.05) (Fig. 4D and G). N1 stage’s risk score was higher than N0 stage’s (*p* < 0.05) (Fig. 4E). M1 stage had higher risk score than M0 stage (*p* < 0.05) (Fig. 4F). We can see the SRlncRNAs signature have trongle relationship to tumor grades, T stages, N stages, M stages, and tumor stages in ccRCC.

**Fig. 5:**
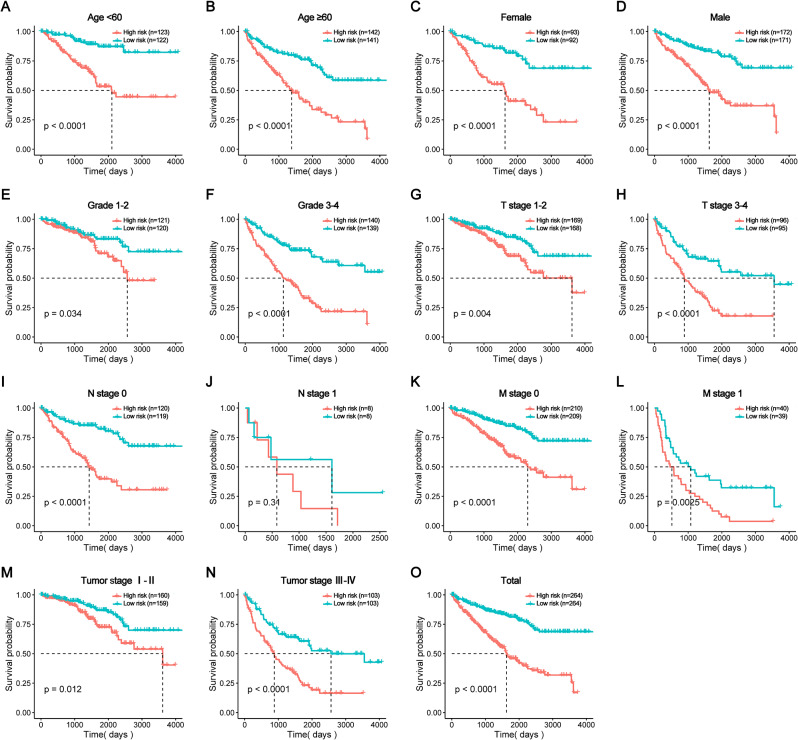
The impact of risk score on ccRCC patients’ prognosis in clinical subgroups. (A-H, K-N) In all different clinical subgroups except N stage, in comparison to patients in low risk groups, those in high risk groups had a decreased survival probability. (I and J) Among different N stage groups, patients’ survival probability was lower in high than low risk, the difference in N 0 stage was significant, but the difference in N 1 stage was not significant. (O) In all patients, the survival productivity was lower in high than the low risk groups. * *p* < 0.05, * * *p* < 0.01, * * * *p* < 0.001

We compared patients’ survival probabilities between high and low risk groups in each clinical subgroup. In all different clinical subgroups except N stage, patients’ survival probability were lower in high than low risk groups (*p* < 0.05) (Fig. 5**A**-**H**, **K**-**N**). In the N0 stage, patients’ survival probability were lower in high than low risk group (*p* < 0.05) (Fig. 5**I**). In N1 stage, patients’ survival probability were also lower in high than low risk group, but the difference had no significance (*p*>0.05) (Fig. 5J). As far as in total patients, the survival probability was lower in high than low risk groups(*p* < 0.05) (Fig. 5O).

### Biological pathways in high and low risk groups

**Fig. 6:**
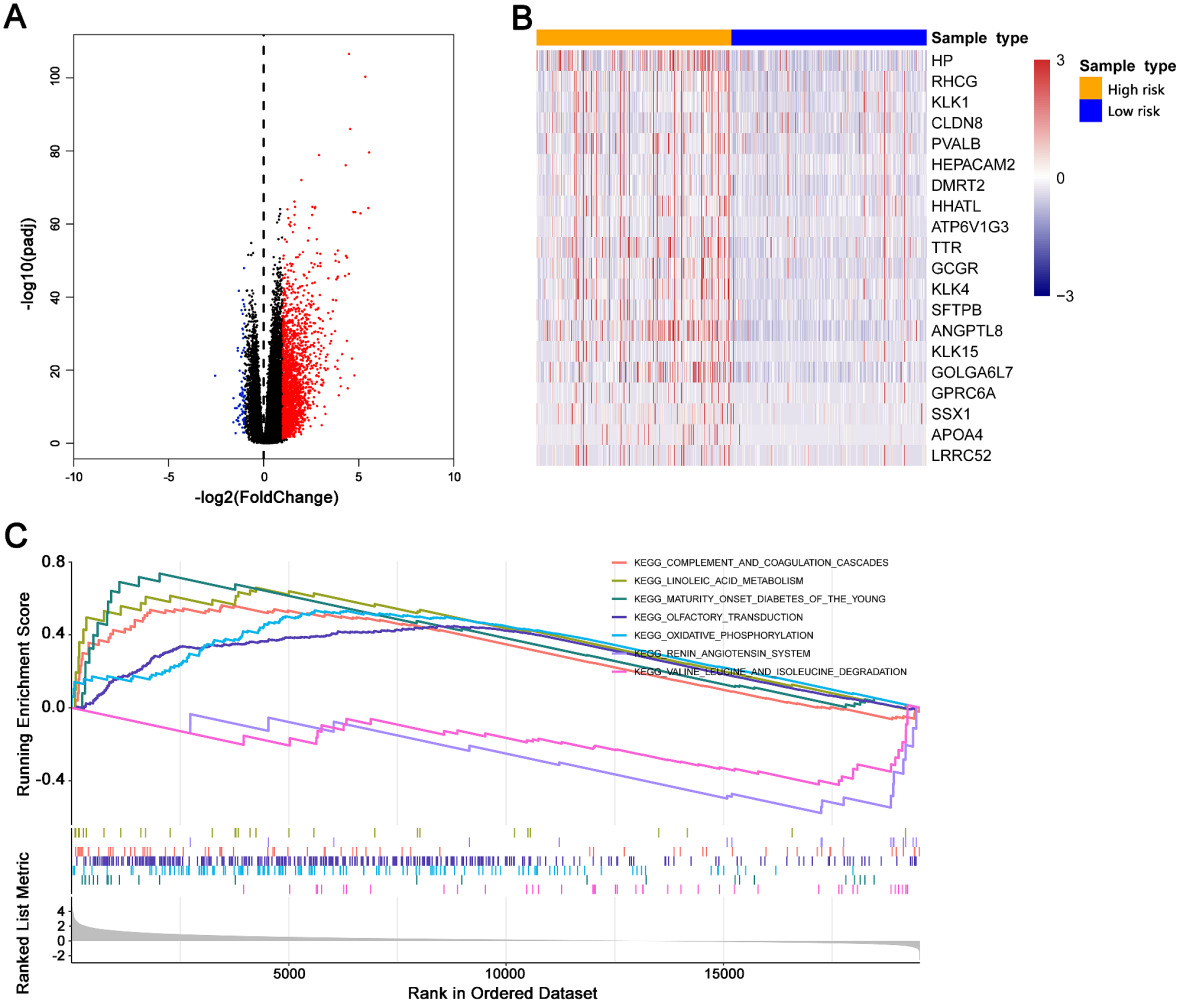
KEGG pathway in high and low risk groups. (**A**) Volcano plot demonstrated the differentially expressed genes in the two groups. (**B**) Top 20 differentially expressed genes. (**C**) The significantly activated and inhibited KEGG pathways in high risk group

For finding differences of biological pathway in the two groups, a gene differential expression analysis was performed firstly (Fig. 6A). KLK1, KLK4, HP, HHATL and RHGG were most differentially expressed in two groups (Fig. 6B). We performed GSEA with the fold changes of all gene, and observed that in high risk group, Maturity-onset diabetes of the young, complement and coagulation cascades, oxidative phosphorylation, linoleic acid metabolism and cytokine-cytokine receptor interaction were significantly activated (p.adjust < 0.05, | NES |>1). In high risk group, degradation of Valine, leucine and isoleucine, and renin-angiotensin system were significantly suppressed (p.adjust < 0.05, | NES |>1) (Fig. 6C).

### The relationship between risk scores and TIME

**Fig. 7:**
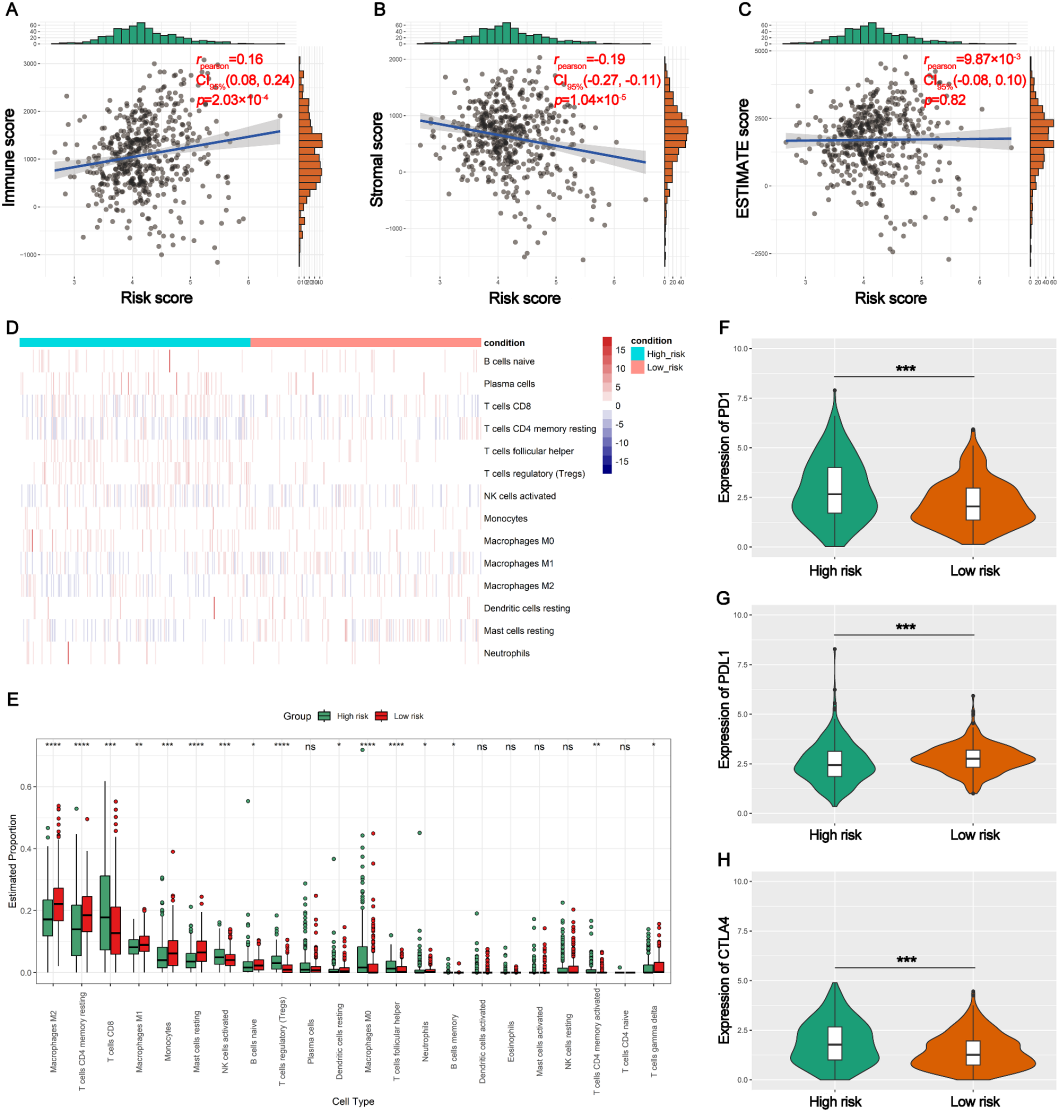
The relationship among the TIME and risk scores in ccRCC. (**A**-**C**) The corraltion of the immune, ESTIMATE and stromal scores with the risk score. (**D**) The heat map demonstrated low and high risk groups’ immune cells infiltration. (**E**) A comparison of the two groups’ immune cell infiltration. (**F**-**H**) A comparison of two groups’ immune checkpoint (PD1, PDL1, and CTLA4) expression. * *p* < 0.05, * * *p* < 0.01, * * * *p* < 0.001

Risk scores had positive correlation with immune scores (*R* = 0.16, *p* < 0.001) (Fig. 7A), negative correlation with stromal scores (*R*=-0.19, *p* < 0.001) (Fig. 7B), no significant correlation with ESTIMATE scores (*R* = 9.87) × 10^− 3^, *p* > 0.05) (Fig. 7C). More activated NK and CD4 memory T cells, regulatory T cells (Tregs), Macrophages M0, memory B cells, follicular helper T cells and CD8 + T cells, in high risk group (*p* < 0.05) than low risk group. The high risk group had less Macrophages M2, gamma delta T cells, Neutrophils, Macrophages M1, Monocells, resetting cells (Mast, Dendritic cells and CD4 memory T) than low risk group (*p* < 0.05) (Fig. 7D and E). Higher expression of PD1 and CTLA4 were observed in high than low risk groups (*p* < 0.05) (Fig. 7F and H). Lower expression of PDL1 was observed in high than low risk groups (*p* < 0.05) (Fig. 7G).

**Fig. 8:**
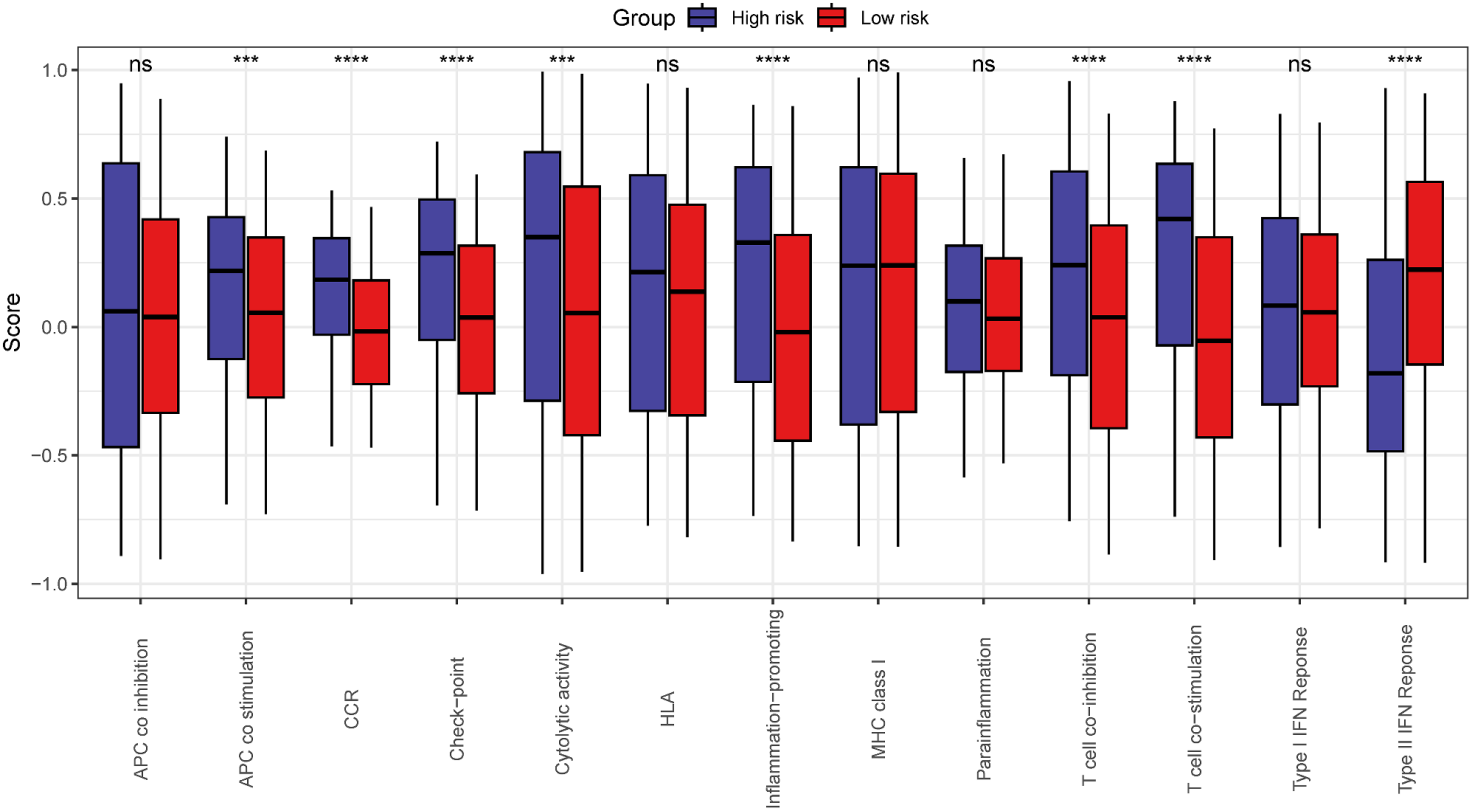
Differences of pathways related immune between high and low risk groups. * *p* < 0.05, * * *p* < 0.01, * * * *p* < 0.001

Compared to the low risk group, T cell co stimulation, Cytolytic activity, Check point, CCR, T cell co inhibition, Inflammation promotion, APC co stimulation were more activated (*p* < 0.05), whereas the high-risk group’s Type II IFN response was more suppressed in the high risk group (*p* < 0.05). APC co inhibition, HLA, Type I IFN Reponse, Parainflamation and MHC class I in high group were not different with low group (*p*>0.05) (Fig. 8).

## Discussion

CcRCC is the commonest type of renal cell carcinoma. The advancements in targeted therapy and immunotherapy have led to a definite improvement in the treatment of ccRCC However, the recurrence and drug resistance leaded numerous patients’ dismal prognosis. Globally, the OS probalility of ccRCC patients has not increased, and the patients with advanced ccRCC suffered the poor quality of life and unfavorable prognosis. Studes indicated that CSCs had a vital impact on the occurrence, advancement, recurrence, tolerance to chemotherapy, and metastasis of RCC [24]. The gene signatures based on tumor stemness is closely associated with the therapy and prognosis of tumors. Researches indicated that lncRNAs could regulate the stemness, pluripotency, drug resistance, and epithelial mesenchymal transition (EMT) of CSCs, and It was highly likely that the lncRNAs become molecular therapeutic targets for tumors [25]. Our study explored the lncRNAs most closely related to the stemness and constructed a SRlncRNAs signature for the prognosis and treatment of ccRNA.

We firstly identified 21 differentially expressed SRlncRNAs closely related to prognose of ccRCC patients. Then, we selected 6 SRlncRNAs to constructed a robust prognostic signature for individuals diagnosed with ccRCC. Finally, we evaluated the signature’s prognostic value in ccRCC patients and its connection with TIME. In our study, internal validation was used to increase SRlncRNAs signature’s predictive accuracy in ccRCC patients prognosis, it was identified that the SRlncRNAs signature was an independent unfavorable predictor for ccRCC individuals’ prognosis, the SRlncRNAs signature was closely related to some biological pathways and TIME of ccRCC.

Metabolism is important for tumor initiation and progression. Studies showed that linoleic acid is closely related to colon cancer and breast cancer [26, 27]. Branched-chain amino acids (BCAA) metabolic reprogramming played an important role in ccRCC [28], Numerous studies showed that decreased BCAA catabolism leads to the advancement of cancer [29, 30]. Oxidative phosphorylation is important to the metastasis and progression of some tumors [31]. In our high risk group, the metabolism of linoleic acid and oxidative physiology were significantly activated, while isoleucine, leucine, and valine degradation were significantly inhibited. The tumor microenvironment is another critical factor affecting numerous tumors’ development. The interaction between cytokine receptors and cytokines, as well as the signaling pathways of chemokines, played an importent role in regulating the immune system [32]. After traumatic organ failure, the activation of the systemic inflammatory response is crucially influenced by the complement and coagulation pathways [33]. By altering the phospholipid membrane of vascular endothelial cells, the interaction between elevated inflammation and complement effectors increases the vulnerability of patients to thrombosis [34]. In our high risk group, the completion and coalescence cascades, cycline and cycline receptor were significantly activated.Erythropoietin (EPO) protein deficiency independently predicts poor RCC prognosis, while intracellular renin (REN) deficiency is an unfavorable prognostic predictor of disease-free survival (DFS) in ccRCC patients, which is associated with venous invasion and high-grade tumor [35, 36]. Our research demonstrated that high risk group have significantly inhibited Renin-Angiotensin System. It can be seen that our SRlncRNAs signature is closely interconnected with tumor metabolism, tumor microenvironment, and Renin angiotensin system.

An important characteristic of ccRCC is the significant infiltration of immune cells, there are lots of Dendritic cells (DCs), CD8 + T cells, macrophages, NK cells and CD4 + T cells [37]. CD8 + T cells were the most important anti-tumor immune cell, the number and anti-tumor activity of these cells had a strong correlation with ccRCC patients’ immunotherapy and prognosis [38]. CD8 + T cells’ anti-tumor activity can be regulated by numerous other immunocytes, including DCs, Tregs, and helper T cells. A study reported that ccRCC secreted cytokines to affect the differentiation of DCs, leading to a reduction or loss of the anti-tumor activity of CD8 + T cells [39]. CD8 + T cells’ activation by mature DCs was linked to better outcomes for patients with ccRCC [40, 41]. M1 macrophages could express IFN- γ, TNF and IL-12 to increase CD8 + T cells’ cytotoxicity [42]. M2 macrophages could collaborate with Tregs to prevent CD8 + T cells to migrate tumor cells [43]. Tregs could inhibite CD8 + T cells’ anti-tumor activity by releaseing TGF- β [44]. NK cells are another important anti-tumor immune cell. It has been reported that IL-2 increased NK cell proliferation and cytotoxicity, which could inhibite the development of ccRCC [45]. A study indicated that a high proportion of NK cells in TIME was linked to the favorable outcome of ccRCC patients [46]. Our research indicated that risk score connected negatively with stromal score and positively with immune score. There were less CD8 + T cells, Tregs, memory B cells, memory CD4 + T cells, Macrophages M0, activated follicular helper T and NK cells in low than high risk group. The high risk group had higher levels of cytological activity, APC co-stimulation, inflammation promotion, checkpoints, CCR, T cell co-inhibition activity and T cell co-stimulation than low risk group. We can see the SRlncRNAs signature is closely related with TIME in ccRCC.

Immunotherapy has become an important treatment method for ccRCC, however its efficacy remains limited to a minority of patients [47]. Research has shown that there is a large amount of CD8 + cell infiltration accompanied by attenuation and functional defects in ccRCC [37]. CD8 + T cells’ activation was suppressed by PD1 and CTLA4, leading to an increase in cellular depletion [48, 49]. A study found that ccRCC patients with poor prognosis had high PD1 expression and a significant amount of CD8 + T cell infiltration [50]. Our study showed that in comparison to the high-risk group, the expression of PD1 and CTLA4 has been reduced in the low-risk group. Therefore, the SRlncRNAs signature may be helpful for forecasting ccRCC patients’ Immunotherapy efficiency.

The SRlncRNAs signature was constructed of six SRlncRNAs including “SNHG17”, “AC007743.1”, “AC114316.1”, “AC108053.1”, “AC103706.1”, “AC068051.1”. As a new cancer related lncRNA, The expression of SNHG17 significantly rises in multiple tumors, suggesting its potential involvement in carcinogenesis. Research has shown that SNHG17 is intimately connected to the growth, invasion, migration, resistance to chemicals, and apoptosis of tumor cells, and head and neck squamous cell carcinoma patients whose SNHG17 expression was high had a significantly unfavorable prognosis [17]. Studies showed that lncRNA AC103706.1 is associated with fat metabolism, copper apoptosis, and prognosis in ccRCC [51, 52]. However, the relationship between six SRlncRNAs and CSCs has not been reported yet.

Nevertheless, there are certain constraints to our research as well. More samples will be beneficial in increasing the accuracy of our results as the TCGA database’s ccRCC sample size is limited. The biological mechanism of SRlncRNAs has not been elucidated through cellular and animal experiments, more research will be conducted in vitro and vivo in the future.

## Conclusions

A reliable prognostic signature was constructed based on six SRlncRNAs for ccRCC patients. The SRlncRNAs signature may be used to indicate the immune microenvironment of ccRCC. It provides a new approach for studying the relationship between lncRNA and CSCs in ccRCC.

## Data Availability

The datasets used in this study are available from the public databases and can be found here: UCSC (https://xenabrowser.net), TCGA (https://portal.gdc.cancer.gov) and Molecular Signatures Database （ https://www.gsea-msigdb.org/gsea/msigdb).
